# Aircraft Noise and Psychological Ill-Health: The Results of a Cross-Sectional Study in France

**DOI:** 10.3390/ijerph15081642

**Published:** 2018-08-03

**Authors:** Clémence Baudin, Marie Lefèvre, Patricia Champelovier, Jacques Lambert, Bernard Laumon, Anne-Sophie Evrard

**Affiliations:** 1 Université Lyon, Université Claude Bernard Lyon1, IFSTTAR, UMRESTTE, UMR T_9405, F-69675 Bron, France; clemence.baudin@ifsttar.fr (C.B.); marie.lefevre@ifsttar.fr (M.L.); 2 IFSTTAR, Planning, Mobilities and Environment Department, Transport and Environment Laboratory, F-69675 Bron, France; patricia.champelovier@ifsttar.fr (P.C.); jamylambert@gmail.com (J.L.); 3 Currently Retired, 69100 Villeurbanne, France; 4 IFSTTAR, Transport, Health and Safety Department, F-69675 Bron, France; bernard.laumon@ifsttar.fr

**Keywords:** epidemiology, aircraft noise exposure, psychological ill-health

## Abstract

*Background:* The effects of aircraft noise on psychological ill-health have not been largely investigated and remain to be discussed. No study has been performed in France on the health effects of aircraft noise. *Objectives:* The present study aimed to investigate the relationship between aircraft noise in dB and in terms of annoyance and psychological ill-health in populations living near airports in France. *Methods:* A total of 1244 individuals older than 18 and living near three French airports (Paris–Charles de Gaulle, Lyon–Saint-Exupéry and Toulouse–Blagnac) were randomly selected to participate in the study. Information about their personal medical history and socioeconomic and lifestyle factors was collected by means of a face-to-face questionnaire performed at their place of residence by an interviewer. Psychological ill-health was evaluated with the 12-item version of the General Heath Questionnaire (GHQ-12). For each participant, outdoor aircraft noise exposure in dB was estimated by linking their home address to noise maps. Objective noise exposure in dB was considered to be the primary exposure of interest. Four noise indicators referring to three different periods of the day were derived and used for the statistical analyses: *L*_den_, *L*_Aeq,24hr_, *L*_Aeq,6hr–22hr_, and *L*_night_. Noise annoyance and noise sensitivity were the secondary risk factors of interest. Logistic regression models were used with adjustment for potential confounders. *Results:* The participation rate in the study was 30%. Approximately 22% of the participants were considered to have psychological ill-health according to the GHQ-12. No direct association was found between exposure to aircraft noise in dB and psychological ill-health. However, annoyance due to aircraft noise and noise sensitivity were both significantly associated with psychological ill-health. Moreover, a gradient was evidenced between annoyance and psychological ill-health, with increasing ORs from 1.79 (95% CI 1.06–3.03) for people who were not all annoyed to 4.00 (95% CI 1.67–9.55) for extremely annoyed people_._ *Conclusions:* These findings confirm the results of previous studies, suggesting there is no direct association between aircraft noise exposure in dB and psychological ill-health, but there is a significant relationship between noise sensitivity or annoyance due to aircraft noise and psychological ill-health. This supports the hypothesis that psychological aspects, such as noise annoyance and noise sensitivity, play important roles in the association between environmental noise and adverse effects on health. However, further studies are necessary in order to better understand the links between these variables.

## 1. Introduction

Transportation noise continues to be a major source of environmental noise pollution and represents a major issue for public health [1]. According to the World Health Organization (WHO), at least one million healthy life years are lost every year due to traffic-related noise in Western Europe [2]. Sleep disturbance and annoyance due to noise are the most serious consequences of environmental noise, mostly related to road traffic [2]. Aircraft noise is the third most important source, after road traffic and railway noise, affecting human exposure above the levels considered to be annoying or to have adverse effects on health [3]. Aircraft noise is perceived as a major environmental stressor near airports. The impact of long-term exposure to aircraft noise on health is of growing concern [4] due to the steady rise in flights as well as the increasing dissatisfaction by nearby inhabitants with this noise [5].

Many studies have demonstrated the adverse effects of exposure to aircraft noise on health, such as annoyance [5,6], sleep disturbance [7,8], cardiovascular diseases including hypertension [9,10,11,12,13], and alteration of cognitive performances among children [14,15]. The association between noise exposure and noise annoyance has been extensively investigated, and aircraft noise has been found to be the most annoying noise source among all transportation noise sources when standardized for noise exposure level [6]. Recently, it has been suggested that annoyance due to aircraft noise has increased in previous years [5,16,17].

In addition, some studies support the hypothesis that psychological aspects such as noise annoyance and noise sensitivity play important roles in the association between environmental noise and adverse effects on health [18,19,20]. Noise is a psychosocial stressor that activates the sympathetic and endocrine systems [21]. As some studies have shown that endocrine distress can lead to psychological symptoms such as depression or anxiety [22,23], the question has been raised as to whether aircraft noise exposure, in dB or in terms of noise sensitivity or noise annoyance, is related to psychological ill-health [24]; however, this has not been largely investigated, and remains to be discussed.

The General Health Questionnaire (GHQ) has been extensively used in large-scale studies for the evaluation of psychological ill-health in the community setting [25]. The four studies investigating the effects of aircraft noise exposure in dB on mental health showed consistent results—they did not find any significant association between aircraft noise exposure and psychological ill-health based on the GHQ-30 [26], the GHQ-28 [27], or the GHQ-12 [28]. Only Miyakawa et al. in Japan showed a significant correlation between aircraft noise exposure and moderate/severe somatic symptoms identified by the GHQ-28 in people sensitive to noise [27]. However, all of these authors observed significant associations between psychiatric illness and noise annoyance [26,28] or noise sensitivity [26,29]. Furthermore, consistent results have been shown regarding the effects of aircraft noise on psychological symptoms, such as depression and anxiety [30], but not for clinically defined psychiatric disorders. Therefore, the effects of aircraft noise on psychological ill-health remain unclear and are still under discussion. Moreover, these effects have never been studied in France and have been investigated by only very few studies in Europe. The study by Tarnopolsky et al. was published in 1980 [26], but aircraft noise levels have changed since the 1980s.

The objective of the DEBATS research program (Discussion on the health effects of aircraft noise) is to investigate the effects of long-term aircraft noise exposure on health among populations living near airports in France. A previous result from the DEBATS study provided support that psychological stress is induced by aircraft noise exposure, resulting in hypothalamus-pituitary-adrenal axis dysregulation and a flattened cortisol rhythm, and notably, a lower ability to decrease cortisol levels at night [31]. The present paper addresses, more specifically, the issue of psychological ill-health among populations living near airports in France, and its association with aircraft noise exposure, annoyance due to aircraft noise and noise sensitivity. The question of whether exposure to high levels of aircraft noise is associated with a higher risk of psychological ill-health is raised.

## 2. Methods

### 2.1. Study Population

The present study included people older than 18 years of age at the time of the interview, living in the study area near one of the following three French international airports: Paris–Charles de Gaulle, Lyon Saint–Exupéry, or Toulouse–Blagnac [11]. The study area was defined based on noise contours produced for France’s largest airports, representing four categories of aircraft noise exposure in terms of *L*_den_: <50, 50–54, 55–59, and ≥60 dB. The *L*_den_ is an annual noise indicator which describes the average equivalent sound pressure levels over a complete year for day (6 a.m. to 6 p.m.), evening (6 p.m. to 10 p.m.), and night (10 p.m. to 6 a.m.) where evening and night sound pressure levels receive a 5 dB and a 10 dB penalty, respectively. The *L*_den_ is the “general purpose” indicator defined in the EU directive 2002/49 relating to the assessment and management of environmental noise. 

Households were randomly selected from a phone directory, based on their address in the study area. Once a household was contacted by phone, a respondent was then randomly selected from within the household. The participant signed and returned an informed consent form by mail. Almost 40% of those contacted who refused to participate responded to a short questionnaire about their demographic and socioeconomic characteristics. It was also possible to compare the characteristics of the participants to those of people who refused to participate (non-participants), as well as to those of the study population, using data from the French national census.

In total, 1244 participants (549 men and 695 women) were included in the study and responded to a questionnaire during a face-to-face interview at their place of residence in 2013. This questionnaire collected demographic and socioeconomic information; lifestyle factors including smoking, alcohol consumption, and physical activity; personal medical history in terms of sleep disturbances, cardiovascular diseases, anxiety, depressive disorders, medication use; and annoyance due to noise exposure. Blood pressure and anthropometric measurements (weight, height, and waist circumference) were also recorded, and saliva samples were taken to determine cortisol levels. 

The analyses presented in the present paper were carried out on the 1222 participants (688 women and 534 men) who had complete information for all the covariates included in the models.

### 2.2. Exposure Assessment

Noise contours are routinely produced by Paris Airports, and the French Civil Aviation Authority for Toulouse–Blagnac and Lyon Saint–Exupéry airports, with the “Integrated Noise Model” (INM) using a height of 4 m for noise simulations [32]. The INM is an internationally well-established computer model that evaluates aircraft noise impacts near airports and outputs noise contours for an area. Outdoor aircraft noise exposure was assessed in 1 dB intervals for each participant with a linkage between the noise contours and their home address using a geographic information system (GIS) technique. Four noise indicators referring to three different periods of the day were derived and used for the statistical analyses: *L*_den_, *L*_Aeq,24hr_, *L*_Aeq,6hr–22hr_, and *L*_night_. The *L*_den_ was used to select the participants (Table 1). The *L*_Aeq,24hr_, *L*_Aeq,6hr–22hr_, and *L*_night_ correspond to the average of sound levels during the corresponding periods of time. 

### 2.3. Psychological Illness

The presence of psychological illness was determined with the 12-item version of the GHQ [33]. The GHQ-12 is a self-reporting instrument for the detection of mental disorders within a community, such as temporary alterations of normal psychological functioning, stable disorders, and stress-related alterations of adaptive behavior. Each of the 12 questions has a four-point response scale, usually scored in a bimodal fashion (respectively 0, 0, 1, 1): ‘not at all’, ‘no more than usual’, ‘rather more than usual’, and ‘much more than usual’. A total score between 0 and 12 was then calculated by summing up the scores of the individual items—the higher the GHQ-12 score, the more psychological distress reported. This total score was then dichotomized in order to determine the presence of psychological ill-health. According to prior studies [34,35,36] and to Goldberg’s recommendations [33,37,38], participants with a total score ≥3 were considered to have psychological ill-health. 

### 2.4. Confounding Factors

The following potential confounders were obtained from the questionnaire with valid and reliable questions used in previous other studies [28,39,40], and introduced into multivariate regression models: gender (dichotomous), age (six categories: 18–34; 35–44; 45–54; 55–64; 65–75; >75 years old), country of birth (two categories: France-born/foreign-born), occupational activity (dichotomous: no/yes), education (three categories: <French high school certificate/French high school certificate/>French high school certificate), marital status (four categories: single/married/widowed/divorced), smoking habits (four categories: non/ex/occasional/daily smoker), alcohol consumption (four categories: no/light/moderate/heavy drinker), number of work-related stress and major stressful life events (three categories: 0/1/more than 2), household monthly income (three categories: <2300; 2300–4000; ≥4000 euros), sleep duration (five categories: ≤5 h; 6 h; 7 h; 8 h; ≥9 h), antidepressant use (two categories: no/yes), and self-reported anxiety (two categories: extremely/a lot versus moderately/slightly/not at all). 

Other a priori confounders, such as house characteristics (window opening, insulation of roof and/or windows) or personal medical history (cardiovascular or other physical diseases) were also initially considered. However, as they were not associated with psychological ill-health in the univariate analysis (*p* > 0.20), they were not included in the multivariate analysis.

Noise sensitivity and annoyance due to aircraft noise were the secondary risk factors of interest. Noise sensitivity was assessed using the following question: “Regarding noise in general, compared to people around you, do you think that you are: less sensitive than, or as sensitive as, or more sensitive than people around you?” Aircraft noise annoyance was assessed by a standardized question with a verbal five-point answer scale as recommended by the International Commission on the Biological Effects of Noise (Icben): “Thinking about the last 12 months when you are at home, how much does aircraft noise bother, disturb or annoy you?” There were five possible answers: extremely, very, moderately, slightly or not at all.

### 2.5. Statistical Analysis

Associations between psychological ill-health and aircraft noise in terms of dB, noise sensitivity or noise annoyance were assessed with logistic regression models. The M0 model included only aircraft noise exposure in dB as an explanatory variable. The M1 model included aircraft noise exposure in dB as the primary exposure of interest, together with major potential confounders as covariates. The M2 model included aircraft noise exposure in dB as the primary exposure of interest, as well as noise sensitivity and noise annoyance as the secondary risk factors of interest, together with confounders. Interactions between noise sensitivity and aircraft noise exposure, annoyance and aircraft noise exposure, and annoyance and noise sensitivity were analyzed in the M2 model.

The linearity of the relationship between the dependent variable and aircraft noise exposure was tested using generalized additive models, including a smooth cubic function with linear and quadratic terms for aircraft noise exposure [41]. As the quadratic term was not significant in these models, associations with the continuous exposure variable were finally estimated per 10 dB increase and are presented in this paper.

All the statistical analyses were performed with SAS 9.3 (SAS Software [program] 9.3 version. USA: Cary, NC, USA, 2011). 

### 2.6. Ethics Approval

Two national authorities in France, the French Advisory Committee for Data Processing in Health Research and the French National Commission for Data Protection and the Liberties approved the present study.

## 3. Results

Overall, the participation rate was 30% (1244 participants/4202 eligible people). Participation rates differed among populations situated near the three airports: 25% for Paris–Charles de Gaulle airport, 34% for Toulouse–Blagnac airport, and 39% for Lyon–Saint-Exupéry airport. In contrast, similar numbers of participants from the four 5 dB-categories of aircraft noise exposure were included. The demographic and socioeconomic characteristics were quite similar among participants, people who refused to participate but responded to the short questionnaire (non-participants), and the study population (Table 1); the participants were a little older and were more likely to have executive or superior intellectual occupations. 

The prevalence of psychological ill-health based on the GHQ-12 was 22% (17% in men and 25% in women). Table 2 shows the odds ratios (ORs) and their 95% CIs for psychological ill-health in relation to levels of aircraft noise in dB and the confounders used in the univariate analysis. The percentage of participants with psychological ill-health did not differ across the four categories of aircraft noise exposure. Women (compared to men), 45 to 54-year-old participants (compared to 18–34-year-old participants), foreign-born participants (compared to France-born participants), daily smokers (compared to non-smokers), people who reported two stressful life events or more (compared to people with no event), people with a household monthly income lower than 2300 euros (compared to people with a household monthly income higher than 4000 euros), and participants who reported anxiety had a higher risk of psychological ill-health according to the GHQ-12. Noise sensitivity and annoyance due to aircraft noise were also significantly associated with psychological ill-health—people who described themselves as more sensitive to noise than others and people who were moderately, very, or extremely annoyed by aircraft noise had a higher risk of psychological distress, as evaluated with the GHQ-12. 

The ORs and their 95% CIs evaluated with the GHQ-12 for psychological ill-health in relation to aircraft noise exposure in three different models (M0, M1 and M2) are presented in Table 3. These analyses involved 1222 participants (688 women and 534 men). They were performed separately for the four noise indicators (*L*_den_, *L*_Aeq,24hr_, *L*_Aeq,6hr-22hr_ and *L*_night_), but as the results were similar between all noise indicators, they are shown for *L*_den_ only. No relationship was observed between aircraft noise exposure in dB and psychological distress, regardless of the noise indicator and the inclusion of confounding factors in the models (M0 and M1 models). When noise sensitivity and annoyance due to aircraft noise were both included in the model (M2 model), there was still no association between psychological ill-health and aircraft noise exposure in dB, regardless of the noise indicator. In contrast, relationships were shown between annoyance due to aircraft noise and psychological ill-health, and between noise sensitivity, and psychological ill-health. Moreover, a gradient was observed between annoyance due to aircraft noise and psychological ill-health; ORs ranged from 1.79 (95% CI 1.06–3.03) for people who were not all annoyed to 4.00 (95% CI 1.67–9.55) for extremely annoyed people. 

Finally, no significant interactions were observed between the noise indicators, noise sensitivity or annoyance due to aircraft noise.

## 4. Discussion

The DEBATS study is the first in France and one of only very few in Europe to investigate the relationship between long-term aircraft noise exposure and psychological ill-health in populations living near airports. The participation rate (30%) was similar to aircraft noise studies completed in Germany, Italy, and in the UK [12]. The prevalence of psychological ill-health evaluated by the GHQ-12 was 22% (17% among men and 25% among women). In contrast, in a Spanish study by Rocha et al., the prevalence of common mental disorders assessed with the GHQ-12 was 30% in women and 17% in men [34]. Further, in a study around Schiphol airport in Amsterdam, carried out in 2005 by van Kamp et al., the prevalence of self-reported mental health complaints evaluated with the GHQ-12 was 26% [28].

The results of the present study confirm those found in the literature, namely that there was no significant association between aircraft noise exposure in dB and psychological ill-health identified with the GHQ-12. However, our findings suggested a gradient between annoyance due to aircraft noise and psychological ill-health, with increasing ORs from 1.79 (95% CI 1.06–3.03) for people who were not all annoyed to 4.00 (95% CI 1.67–9.55) for extremely annoyed people. Miedema and Oudshoorn [6] showed evidence for a dose–response relationship between aircraft noise exposure and the percentage of highly annoyed people. These exposure–response relationships are used as the standard curves for the assessment and management of environmental noise in the European Union [42]. Therefore, it could be assumed that an increase in aircraft noise exposure leads to an increase in annoyance due to aircraft noise, thus leading to an increase in psychological ill-health. However, further research is necessary to validate this hypothesis.

One of the first studies to assess the effects of aircraft noise on mental health was performed by Tarnopolsky et al. in 1980 [26]. Although the authors did not observe any excess psychiatric morbidity identified by the GHQ-30 in populations exposed to aircraft noise, they showed an association between psychiatric illness and noise annoyance or sensitivity to noise. In the longitudinal study around Schiphol airport in Amsterdam [28], which is the most similar to the DEBATS in terms of methodology, the authors did not observe any association between noise exposure levels or changes in exposure levels after the opening of the fifth runway and mental health complaints as measured by the GHQ-12 (OR = 0.94 for a 3 dB-increase in noise levels in terms of *L*_den_, 95% CI = 0.84–1.05). However, people who were severely annoyed by aircraft noise reported more mental health complaints, as assessed by the GHQ-12 (OR = 1.84, 95% CI = 1.38–2.45). In Japan, Miyakawa et al. [27] did not observe any relationship between aircraft noise exposure and psychiatric disorders evaluated with the GHQ-28 but showed a significant correlation between aircraft noise exposure and moderate/severe somatic symptoms in people sensitive to noise. In Spain, outside noise reported as a perceived environmental problem was significantly associated with the prevalence of common mental disorders using the GHQ-12 [34]. Finally, in the United Kingdom, high noise sensitivity was identified by Stansfeld et al. [29] as a predictor of psychological distress using the GHQ-30. 

In the present study, a relationship was observed between noise sensitivity and psychological ill-health, and between annoyance due to aircraft noise and psychological ill-health, irrespective of noise exposure. Both relationships were significant, underlining the independent effects of both factors and supporting the hypothesis that psychological aspects such as noise annoyance and noise sensitivity seem to play important roles in the association between environmental noise and adverse effects on health. 

On one hand, it has been postulated that, if a (direct) relationship does not exist between noise exposure in dB and psychological ill-health, annoyance may be regarded as an intermediate step in the causal chain between aircraft noise exposure and health, in particular, psychological ill-health. However, the relationship between noise annoyance and psychological ill-health is still under discussion. Because of the cross-sectional design of major studies, the direction of the association has been questioned. Extremely annoyed people might be more at risk of having psychological ill-health, but it is also possible that people with psychological ill-health might be more at risk of being annoyed and then be more willing to attribute their symptoms to noise [19,20,43]. However, it was not possible to answer this question in the present study.

On the other hand, noise sensitivity is considered as a moderating factor of the effects of aircraft noise exposure on noise annoyance [18,44]. It has been suggested that noise sensitivity could also influence the effects of noise on physical and psychological ill-health [45]. Noise sensitivity has been suggested to be a potential indicator of vulnerability to environmental stressors, not only to environmental noise [46,47], it has also been postulated to be a proxy measure of anxiety [29]. However, further research is necessary to better understand how noise sensitivity and psychological ill-health are linked.

A specific strength of the present study relates to the evaluation of noise exposure. Outdoor aircraft noise exposure was estimated for each participant with modeled noise levels produced by the French Civil Aviation Authority using INM software. Most of the differences between these modeled noise levels and measurements from permanent stations [48] or from specific campaigns [49] were between 0.5 and 1.5 dB in terms of *L*_den_, showing the close correspondence between modeled and measured noise levels. 

In terms of limitations, aircraft noise exposure was estimated in front of each participant’s residence. Nevertheless, this estimation did not take into account the building outdoor insulation and the opening/closing practice of the windows, thus leading to a potential misclassification of the participants according to their noise levels. Moreover, many of the participants, at least those who were at work, were more likely to be away from their homes during the day. No information was available about the daytime aircraft noise exposure of the participants when they were away from their homes, for example, at their workplace. Thus, misclassification of exposure could have occurred, especially regarding daytime exposure. However, it is unlikely that the exposure classification would depend on the psychological distress of the participants. Therefore, such non-differential misclassification would have induced an appreciable downward bias if there is a true association between aircraft noise exposure and psychological ill-health, thus explaining the absence of an association observed in the present study. 

Furthermore, a selection bias cannot be excluded in the present study. Participants were slightly different from people who refused to participate but responded to the short questionnaire, particularly in regards to their age and their socio-occupational category. In addition, these non-participants were not representative of all people who refused to participate. The representativeness of a sample randomly selected from a phone directory (certainly with a better socioeconomic situation than that of the study population) could be raised but could not be quantified in the present study. The same applies for the representativeness of the study population as compared with all people living near an airport in France. However, due to insufficient information, it was not possible to characterize this latter population. 

Another form of selection bias may have occurred during the estimation of the prevalence of psychological ill-health. This prevalence may have been underestimated in the higher noise zones if unsusceptible individuals were selected in these zones. The possible adverse effects of aircraft noise on psychological ill-health could have led to a lower proportion of sensitive people among those living near airports, particularly in the higher noise zones. People prone to illness, especially to psychological ill-health, may be reluctant to live in noisy conditions. Little information is available in the DEBATS study to judge whether people with psychological problems have chosen not to live close to airports. However, if this had occurred, it would have resulted in an underestimation of the association between aircraft noise exposure and psychological ill-health in this study. It is therefore possible that a background of better mental health in the higher noise zones could hide noise effects on psychological ill-health in this study. 

It is unlikely that a lack of statistical power caused the failure of the present analysis to find a significant association between aircraft noise exposure in dB and psychological ill-health. Indeed, the number of participants included in the DEBATS study (*n* = 1244) was very significant. Other studies did not observe any association in this regard, despite a higher number of participants and thus greater statistical power: 2671 people were included in the study by van Kamp et al. [28], and 2861 in the one by Miyakawa et al. [27]. Moreover, a significant association was previously shown between aircraft noise exposure and a smaller variation in cortisol levels among the participants in the DEBATS study [31]. This finding provides some support for a link between psychological stress and aircraft noise exposure, and, as endocrine distress could lead to psychological symptoms such as depression or anxiety [22,23], it suggests a method by which aircraft noise exposure could cause psychological ill-health. Nevertheless, such an association was not observed in the present analysis. 

A more appropriate indicator of psychological distress than the GHQ might show a relationship with aircraft noise exposure in dB. The fact that psychological ill-health was estimated using a questionnaire could be a limitation in the present study although it has been used by most previous studies on psychological illness [26,27,28,29,34,50]. The GHQ-12 is a reliable screening questionnaire that is particularly recommended for identifying minor psychological disorders within community settings. Since the GHQ-12 is brief, simple, easy to complete, and its application in research settings as a screening tool is well documented, the GHQ-12 has been widely used in large-scale studies in the way that it can serve as a general indicator of distress. Nevertheless, it is not a tool for indicating a clinical diagnosis. Moreover, the double dichotomization (of the response scale by using the bimodal scoring method and of the total score by considering participants with a total score ≥3 as having psychological ill-health) raised the question of the sensitivity of the scale measuring psychological disorders. However, the results remained similar when the four-point response scale of the 12 questions was scored using the Likert scoring method (0, 1, 2, 3, respectively) or when linear regression models with the total score as a continuous outcome variable were used. Prescribed and non-prescribed medication could also be used as proxies to characterize mental health. For example, the largest study to date, which included around six major European airports—the HYpertension and Exposure to Noise near Airports (HYENA) study—found that a 10 dB increase in day-time (*L*_Aeq,_ _6hr–22hr_) or night-time (*L*_night_) aircraft noise was associated with a 28% increase in anxiety medication use, but not with anti-depressant medication use [51]. Information about prescribed and non-prescribed medication taken by the participants was also collected in the present study. The results presented here considered anti-depressant medication to be a confounding factor but they remained unchanged when this variable was not introduced in the models. Further research is necessary to better understand the relationships between aircraft noise exposure and medication use (including anti-depressant use).

Only a standardized clinical interview including questions about the number and the severity of symptoms can measure psychiatric disorders, but this can be expensive and time consuming for large-scale epidemiological studies and the response rate may be low. In the last few years, some epidemiological studies have tried to investigate mental health based on clinical diagnosis and average noise exposure—both from road traffic and airport noise. In Germany, Orban et al. suggest that exposure to residential road traffic noise increases the risk of depressive symptoms [52]. A large case-control study in the region of Frankfurt international airport by Seidler et al. indicates that traffic noise exposure—from aircraft, road traffic, and railway—might lead to depression [53]. However, further prospective research is needed to confirm the results of these studies and to deepen knowledge of the causal pathway between noise exposure and depression.

## 5. Conclusions

The DEBATS study is the first in France and one of only very few in Europe to investigate the relationship between long-term aircraft noise exposure and psychological ill-health in populations living near airports. The results of this study are consistent with those found in the literature, suggesting no association between aircraft noise exposure in dB and psychological ill-health evaluated with the GHQ, but showing an association between noise sensitivity or annoyance due to aircraft noise and psychological ill-health. In addition, a gradient was shown between annoyance due to aircraft noise and psychological ill-health. These findings support the hypothesis that psychological aspects such as noise annoyance and noise sensitivity play important roles in the association between environmental noise and adverse effects on health. Nevertheless, further research is needed to disentangle the possible effects of noise, sensitivity to noise, and annoyance due to noise on psychological ill-health, as well as how these factors are linked. 

## Figures and Tables

**Table 1:** Comparison of the demographic and socioeconomic characteristics of participants, non-participants, and the study population.

	Participants		Non-Participants ^1^		Study Population ^2^
	*n*	%	*n*	%	%
**Noise level (*L*_den_ in dB)**					
**Paris-Charles de Gaulle**					
<50	108	17%	324	22%	-
50–54	102	16%	215	14%	-
55–59	208	34%	464	31%	-
≥60	202	33%	497	33%	-
**Toulouse-Blagnac**					
<50	104	25%	198	29%	-
50–54	103	25%	159	23%	-
55–59	101	25%	160	23%	-
≥60	103	25%	169	25%	-
**Lyon Saint-Exupery**					
<50	105	49%	166	57%	-
50–54	102	48%	124	43%	-
55–59	5	2%	1	0%	-
≥60	1	1%	0	0%	-
**Gender**					
Men	549	44%	1028	41%	48%
Women	695	56%	1449	59%	52%
**Age**					
18–34	226	18%	497	20%	26%
35–44	236	19%	435	18%	17%
45–54	266	21%	416	17%	19%
55–64	260	21%	448	18%	15%
65–74	185	15%	332	13%	13%
≥75	71	6%	331	13%	10%
**Marital status**					
Single	253	20%	555	22%	-
Married	782	63%	1326	54%	-
Widowed	76	6%	281	11%	-
Divorced	133	11%	194	8%	-
Other	0	0%	10	0%	-
Unknown/refusal	0	0%	111	5%	-
**Socio-occupational category**					
Farming, trade	32	2%	81	3%	5%
Executive, superior Intellectual occupation	227	18%	322	13%	9%
Intermediate	220	18%	103	4%	14%
Office worker	268	22%	749	30%	17%
Manual worker	79	6%	145	6%	13%
Retiree	337	27%	929	38%	25%
Never worked or long-term unemployed (students, housewives, other)	81	7%	134	5%	17%
Unknown/refusal	0	0%	14	1%	-

^1^ People randomly selected and contacted by phone, but who refused to participate. These people responded to a short questionnaire about their demographic and socioeconomic characteristics. ^2^ The distribution of the study population is based on data from the 1999 INSEE census, adjusted in 2007, for individuals aged 18 and over and living in one of the 161 municipalities of the study area.

**Table 2:** Odds ratios (ORs) for psychological ill-health in relation to major confounders in univariate logistic models.

	N	Number of Participants with GHQ-12 ≥ 3	Number of Participants with GHQ-12 < 3	OR	(95% CI)
**Noise levels (*L*_den_ in dB)**					
<45	82	25 (30%)	57 (70%)	1	-
45–49	235	49 (21%)	186 (79%)	0.60	(0.34–1.06)
50–54	307	62 (20%)	245 (80%)	0.58	(0.33–1.00)
55–59	314	66 (21%)	248 (79%)	0.61	(0.35–1.04)
≥60	306	66 (22%)	240 (78%)	0.63	(0.36–1.08)
**Noise sensitivity**					
As sensitive or less sensitive than people around you	866	154 (18%)	712 (82%)	1	-
More sensitive than people around you	369	111 (30%)	258 (70%)	**1.99**	**(1.50–2.64)**
**Annoyance due to aircraft noise**					
Not at all annoyed	246	37 (15%)	209 (85%)	1	-
Slightly	312	65 (21%)	247 (79%)	1.49	(0.95–2.32)
Moderately	460	99 (22%)	361 (78%)	**1.55**	**(1.02–2.34)**
Very	186	50 (27%)	136 (73%)	**2.08**	**(1.29–3.35)**
Extremely	40	17 (43%)	23 (57%)	**4.18**	**(2.04–8.56)**
**Gender**					
Men	549	92 (17%)	457 (83%)	1	-
Women	695	176 (25%)	519 (75%)	**1.68**	**(1.27–2.23)**
**Age**					
18–34	226	43 (19%)	183 (81%)	1	-
35–44	236	58 (25%)	178 (75%)	1.39	(0.89–2.16)
45–54	266	71 (27%)	195 (73%)	**1.55**	**(1.01–2.38)**
55–64	260	56 (22%)	204 (78%)	1.17	(0.75–1.82)
65–74	185	26 (14%)	159 (86%)	0.70	(0.41–1.18)
≥75	71	14 (20%)	57 (80%)	1.05	(0.53–2.05)
**Country of birth**					
France-born	1054	215 (20%)	839 (80%)	1	-
Foreign-born	190	53 (28%)	137 (72%)	**1.51**	**(1.06–2.14)**
**Occupational activity**					
No	499	100 (20%)	399 (80%)	1	-
Yes	745	168 (23%)	577 (77%)	1.16	(0.88–1.53)
**Education**					
<French high-school certificate	452	97 (21%)	355 (79%)	1	-
French high-school certificate	215	52 (24%)	163 (76%)	1.17	(0.79–1.72)
>French high-school certificate	577	119 (21%)	458 (79%)	0.95	(0.70–1.29)
**Marital status**					
Single	253	56 (22%)	197 (78%)	1	-
Married	782	162 (21%)	620 (79%)	0.92	(0.65–1.3)
Divorced	133	34 (26%)	99 (74%)	1.21	(0.74–1.97)
Widowed	76	16 (21%)	60 (79%)	0.94	(0.50–1.75)
**Smoking habits**					
Non-smoker	625	120 (19%)	505 (81%)	1	-
Ex-smoker	330	74 (22%)	256 (78%)	1.22	(0.88–1.69)
Occasional smoker	19	1 (5%)	18 (95%)	0.23	(0.03–1.77)
Daily smoker	269	72 (27%)	197 (73%)	**1.54**	**(1.10–2.15)**
**Alcohol consumption**					
No	348	89 (26%)	259 (74%)	1	-
Light	637	134 (21%)	503 (79%)	0.78	(0.57–1.05)
Moderate	193	31 (16%)	162 (84%)	**0.56**	**(0.35–0.88)**
Heavy	54	10 (19%)	44 (81%)	0.66	(0.32–1.37)
**Number of work-related stress and major stressful life events**					
0	287	46 (16%)	241 (84%)	1	-
1	330	57 (17%)	273 (83%)	1.09	(0.71–1.67)
≥2	627	165 (26%)	462 (74%)	**1.87**	**(1.30–2.69)**
**Household monthly income**					
≥4000 euros (4500 US$)	319	56 (18%)	263 (82%)	1	-
2300–4000 euros (2600–4500 US$)	474	93 (20%)	381 (80%)	1.15	(0.79–1.65)
<2300 euros (2600 US$)	451	119 (26%)	332 (74%)	**1.68**	**(1.18–2.40)**
**Sleep duration**					
≤5 h	52	9 (17%)	43 (83%)	0.65	(0.31–1.40)
6 h	256	30 (19%)	126 (81%)	0.74	(0.47–1.18)
7 h	363	88 (24%)	275 (76%)	1	-
8 h	424	94 (22%)	330 (78%)	0.89	(0.64–1.24)
≥9 h	249	47 (19%)	202 (81%)	0.73	(0.49–1.08)
**Antidepressant use**					
No	1203	255 (21%)	948 (79%)	1	-
Yes	41	13 (32%)	28 (68%)	1.73	(0.88–3.38)
**Self-reported anxiety**					
Moderately/slightly/not at all	978	122 (12%)	856 (88%)	1	-
Extremely/a lot	266	146 (55%)	120 (45%)	**8.54**	**(6.28–11.61)**

**Table 3:** Odds ratios (ORs) for the relationship between aircraft noise exposure and psychological ill-health.

	OR	(95%CI)
**M0 Model**		
***L*_den_^1^**	0.91	(0.72–1.14)
**M1 Model**		
***L*_den_^1^**	1.02	(0.78–1.34)
**M2 Model**		
***L*_den_^1^**	0.93	(0.69–1.24)
**Noise sensitivity**		
Less or as sensitive as people around you	1.00	
More sensitive th. people around you	**1.52**	**(1.09–2.14)**
**Annoyance due to aircraft noise**		
Not at all annoyed	1.00	
Slightly	**1.79**	**(1.06–3.03)**
Moderately	1.63	(0.98–2.71)
Very	**2.00**	**(1.10–3.64)**
Extremely	**4.00**	**(1.67–9.55)**

^1^ Per 10 dB increase. M0 = Univariate regression model including only aircraft noise exposure in terms of *L*_den_. M1 = Multivariate regression model including aircraft noise exposure in terms of *L*_den_ together with the major potential confounders listed in Table 2 (without noise sensitivity and annoyance due to aircraft noise). M2 = Multivariate regression model including aircraft noise exposure in terms of *L*_den_ together with noise sensitivity, annoyance due to aircraft noise and the major potential confounders listed in Table 2. Bold values are statistically significant (*p* < 0.05).
